# The Effectiveness of Culturally Specific Male Domestic Violence Offender Intervention Programs on Behavior Changes and Mental Health: A Systematic Review

**DOI:** 10.3390/ijerph192215180

**Published:** 2022-11-17

**Authors:** Lata Satyen, Ashlyn Hansen, Jane Louise Green, Laura Zark

**Affiliations:** School of Psychology, Deakin University, Burwood, VIC 3125, Australia

**Keywords:** domestic violence, perpetrator, intervention, behavior modification, mental health, culture, ethnicity

## Abstract

The objective of domestic violence intervention programs is to address perpetrator behavior. However, the suitability and effectiveness of these programs in confronting problematic behavior for ethnically diverse groups is unclear. Therefore, the aim of this systematic review was to cohesively examine whether such programs are effective in reducing recidivism, changing perpetrator behavior, and addressing mental health issues for culturally diverse groups. Several databases were searched for peer-reviewed articles that included culturally specific components or ethnically diverse cohorts in offender intervention programs. 10 articles met the inclusion criteria. The findings demonstrate greater effectiveness of programs with greater cultural engagement: through culturally trained facilitators, addressing the cultural and patriarchal norms relevant to the specific client group, and discussion of gender roles and attitudes to gender equality specific to the cultural context. Such programs achieved some positive outcomes including: reduced recidivism, improved mental health, and better attitudes to gender equality. However, the findings are limited to a few ethnically diverse groups, and not all studies measured all outcomes listed above. This review suggests the development and implementation of suitable offender intervention programs that address perpetrator behavior and mental health in ethnically diverse client groups. When culturally relevant and effective programs are implemented, it could lead to men’s modification of perpetrating behavior and create safer family relationships.

## 1. Introduction

With the ongoing increase in rates of violence against women, it is necessary to develop effective interventions to change perpetrator behavior. This violence continues despite the range of policies, prevention and intervention programs, and funding allocated to address the issue. We know that globally, one in four ever-partnered women have experienced physical or sexual violence from a male intimate partner since the age of 15 years [1]. There is also evidence that some communities are at higher risk of women experiencing violence from a male intimate partner, with the highest prevalence rate in low-income regions [1,2]. These regions tend to have populations with increased cultural diversity and ethnic minority groups, and associated intersectional risk factors including trauma, discrimination, social and economic deprivation, social isolation and pre and post-migration stress [3]. The violence is caused by individual factors and social systems that influence perpetrators to commit physical, psychological, sexual, financial, spiritual, social abuse [4], and a pattern of coercive control. Given the serious and long-lasting physical, social, and mental health consequences of intimate partner violence (IPV), the World Health Organization (WHO) [5] has called for higher quality intervention and prevention strategies to reduce the prevalence and impact of IPV. While acknowledging that IPV occurs in diverse partnerships, this review focuses on male perpetration towards their female partners. While no community is showing decreasing levels of violence against women, we need to examine the effectiveness of existing violence prevention and intervention programs in reducing rates of violence in cultural minority and non-Anglo-Saxon groups. This will help us identify universal and unique factors that can be implemented to achieve the goal of behavior change to reduce recidivism rates, improve mental health and ensure women are able to live in a safer society.

### 1.1. Interventions for Perpetrators

A range of mechanisms have been developed to address male perpetration of intimate partner violence. The most commonly used perpetrator intervention strategies to date include mandatory arrest, feminist sociocultural programs (e.g., the Duluth model) [6], and cognitive-behavioral therapy (CBT) [7]. Feminist sociocultural programs focus on addressing gender-related attitudes, encouraging accountability and personal responsibility for abusive behavior, and promoting gender-equal attitudes and behaviors [8]. The emphasis placed by feminist sociocultural programs on male perpetrators’ choice to be violent means that to benefit from these programs, perpetrators must be motivated to alter their notions of power and control [9]. However, a recent, comprehensive review asserted that if perpetrators are unwilling to actively participate in programs to alter violent behavior, feminist principles may be of little use in facilitating change [10]. We therefore need to examine alternate approaches to maladaptive patterns of thinking.

A few programs focus on psychotherapy and identifying individual causes of violence including behavioral deficits or psychopathology [11]. Although CBT can address individual behavioral issues, most CBT-related perpetrator programs are administered in a group setting that focus on skills training for anger management, better communication, assertiveness, and relaxation [8]. Further, CBT assumes that clients are motivated to change their behavior and capable of altering their cognitive or behavioral patterns [12]. However, research shows that many abusive men do not have the capacity to engage in treatment methods, or to alter their cognitions related to power and control, and increase their empathy for their partners and children [13]. The literature therefore suggests that feminist and cognitive-behavioral approaches may be ineffective because men are either not willing or able to change their thinking and/or behavior. This leads us to examine what successes existing CBT-focused behavior intervention programs are achieving.

The Duluth model was originally implemented with feminist and sociocultural underpinnings and using the constructs of power and control [6]; it has since been adapted to apply a combination of cognitive-behavioral techniques and feminist approaches [14]. It aims to both challenge men’s sense of power and control and teach them alternative skills to reduce conflict in their relationships and reduce their perpetration of violence towards their partners [14]. This approach is one of the few that has been applied to male perpetrators of African American, Native American, and Latino descent, apart from Ango-Saxons [10]. In spite of some of its positive facets, it is limited in its acknowledgement of men’s issues related to racism and discrimination; further, it does not address immigration-related challenges and their relationship to perpetration, and has not been evaluated for its effectiveness with other ethnic groups [15,16]. Thus, despite the development and implementation of a range of domestic and family violence perpetrator intervention programs, the adequacy and efficacy of these programs appears to be lacking.

Evaluations of intervention programs have shown that group treatments for IPV perpetrators have limited success in altering the cycle of violence [8,17]. Indeed, irrespective of the strategy adopted, one in three perpetrators exhibit a new abusive pattern of IPV within 6 months of a victim’s report [8]. A few reasons have been elucidated about the challenges to treating this part of the population. Most perpetrators present a combination of being child victims of family violence [18,19] and characteristics such as impulsivity, psychological entitlement, poor regulation of anger, and empathy impairment as well as narcissistic and antisocial behavior [20,21] along with substance abuse problems [22]. Such behavioral risk factors associated with adverse childhood experiences can lead to enduring negative effects on brain development and thus, are difficult to address as part of behavioral modifications [23].

Traditional IPV treatment models based on the principles of CBT or the Duluth model have been shown in systematic reviews to have little to no effect on recidivism [11,24,25], although significant methodological issues and high attrition rates preclude clear conclusions on their effectiveness. These findings also vary considerably across populations and settings [11,24]. Additionally, Chang and Saunders [26] highlight the elevated risk of attrition from these programs amongst minority groups. The majority of research evaluating the effectiveness of these models has been conducted in the North American context [27] and a number of studies have found that these programs may be inappropriate or ineffective for ethnic and cultural minority groups [16,28]. Thus, research demonstrates mixed findings in relation to the adequacy of intervention programs to address perpetrator behavior and reduce the rates of violence against women in the community.

### 1.2. Theoretical Perspectives of Perpetrator Intervention Programs

The theoretical premises on which perpetrator intervention programs have been developed include ecological, cognitive-behavioral, feminist, and intersectional approaches. Ecological approaches [29] attempt to address the individual, relational, and societal level factors that might influence a perpetrator’s abusive behavior; while cognitive-behavioral approaches endeavor to restructure beliefs regarding gender and gendered violence and teach skills for effective communication and self-regulation, particularly with regard to regulation of anger [7]. Feminist-focused intervention programs aim to instill gender-positive attitudes and challenge hierarchical patriarchal structures [9,30]. Programs that utilize an intersectional approach posit that gender-based violence cannot be understood without exploring the interactions between gender inequality and other forms of marginalization, such as those based on race and class (e.g., Crenshaw, 1989) [31]. This approach considers the different experiences of groups who occupy positions of privilege and of marginalization on the basis of multiple intersecting identities [3,10,32,33].

In the context of working with minority ethnic groups and non-Anglo-Saxon communities, program practitioners need to be culturally sensitive and ensure that the impact of culture is not minimized or misunderstood, particularly as most treatment systems are guided by dominant White-centric theories [32]. ‘Culture’ should also not be an “added-on” feature of programs [32]. Rather, practitioners need to develop an in-depth knowledge of cultural norms of the communities they are engaging with, including the practices of dowry, arranged marriage, and female genital mutilation. Importantly, practitioners must not assume that violence against women is “culturally normative” [32].

### 1.3. Ethnicity and Intimate Partner Violence Perpetrator Programs

Research on culturally specific interventions to reduce IPV remains scant [24,34]. These interventions have not been thoroughly evaluated or have been evaluated using different measures of success. Examining the evidence for the use of such programs, and particularly their application in ethnic minority and non-Anglo-Saxon groups, is necessary for developing and implementing appropriate program protocols for these groups. Turhan [10] recently conducted a narrative review of current approaches to IPV perpetrator intervention to evaluate their suitability for marginalized ethnic subpopulations. He found variations in the degree to which ‘culture’ was applied across existing intervention programs. For example, some paradigms associated domestic violence with social issues such as traditional patriarchal and gender roles, spirituality, and immigration-related stressors [32,35] while others [10,36] found structural issues such as practitioners’ knowledge of racism and discrimination to influence the success of the program.

Client-related issues can also impact engagement with an intervention. For example, Turhan [10] found that program participants’ knowledge of the premise of therapy, language barriers, and awareness of the issue of domestic violence could lead to increased program engagement and reduced attrition. The importance of the establishment of rapport and trust between the client and program facilitator were also highlighted as important factors in creating a positive therapeutic environment [10]. This was in addition to practitioners being able to apply appropriate techniques that address men’s needs in their social and cultural contexts [10].

Program developers need to identify and address various risk factors for men from ethnic minority backgrounds. Studies illustrate that hardship, trauma, and psychological distress, as well as acculturation and shifts in cultural beliefs and values, can contribute to family conflict [37,38]. Migration-related stressors such as financial and employment pressures, loss of informal supports, lack of access to health and social services, and exposure to discrimination in the host country may also act as cumulative risk factors for IPV amongst ethnic and cultural minority groups [39,40,41,42,43]. Programs that incorporate ways of discussing these issues and intervening in culturally relevant ways have a greater chance of being successful. Migration-related stressors also call for social justice systems to be more supportive and for communities to create better social supports for families in abusive relationships.

Given the close relationship of ‘culture’ to the constructs of family and gender roles, which are the focus of current IPV treatment models [11,44,45], it is necessary to develop culturally appropriate approaches for IPV perpetrators from ethnic minority and non-Anglo-Saxon groups. A failure to provide culturally informed interventions presents a risk to such groups, who may not benefit from dominant IPV perpetrator treatment models [46]. Indeed, integrative programs that are community-driven and foster culturally sensitive discussions about gender, power, and violence have greater acceptability and effectiveness for promoting non-violent behavior in cultural and ethnic minority communities [45,46,47]. To date, there is no comprehensive review of culturally specific IPV perpetrator programs for ethnic minority and non-Anglo-Saxon populations to measure recidivism and other program outcomes. Therefore, the aim of this review was to systematically examine the evidence for the effectiveness of culturally specific interventions for male perpetrators of domestic and family violence to achieve positive behavior change and address mental health issues.

## 2. Materials and Methods

### 2.1. Search Strategy

Studies were identified by systematically searching thirteen electronic databases for the period 1993–2022, with the final search of all databases being conducted on 9 August 2022: Academic Search Complete, AMED, CINAHL Complete, Criminal Justice Abstracts with Full Text, E-Journals, Health Source: Nursing/Academic Edition, MEDLINE, MEDLINE Complete, PsycARTICLES, PsycEXTRA, Psychology and Behavioural Sciences Collection, PsycINFO, and Social Work Abstracts. The effective combination of search terms was designed and set up by one reviewer according to the PRISMA statement [47] and different terms and rules of each database. Reference lists of retrieved studies were then hand-searched and papers citing these relevant studies in Google Scholar were screened to identify any additional studies that may have been overlooked. Specific keywords and free text terms for perpetrators of intimate partner violence, interventions and culture were used for each database (Supplementary Materials S1). Only studies with key terms in their title or abstract were included for review.

### 2.2. Study Selection

Any peer-reviewed journal article that evaluated the effectiveness of culturally specific interventions for adult male perpetrators of intimate partner violence and was written in the English language and published between January 1993–August 2022, was considered for review. Studies were excluded if any participants were identified as female or under the age of 18 years; or if the intervention program was not culturally specific. Furthermore, studies that did not measure outcomes related to the effectiveness of the intervention, such rates of recidivism and re-assaults; changes to psychopathological symptoms related to perpetration including anger, empathy, impulsivity, self-esteem and inadaptation; attitudes towards gender and sexuality; and satisfaction with intervention engagement, were excluded. Studies not published in the English language were also excluded.

### 2.3. Screening and Data Extraction

One researcher (A.H.) screened titles and abstracts of studies which were identified through electronic database searches, following the removal of duplicate references using EndNote X8.2 software (Clarivate, London, UK). Full text articles of potentially relevant studies were then assessed independently by three co-authors (A.H., L.S. and L.Z). Discrepancies in eligibility assessment were resolved through discussion between all four co-authors.

Key study characteristics and outcomes were then extracted as indicated in Supplementary Materials S2. Study characteristics included: risk of bias, country of participant population, inclusion and exclusion criteria, demographics of participants, sample size, format and description of culturally specific intervention component, type and description of comparison condition. Study outcomes extracted included: intervention effect on recidivism and re-assaults; changes to psychopathological symptoms related to perpetration including anger, empathy, impulsivity, self-esteem and inadaptation; attitudes towards gender and sexuality; and satisfaction with intervention engagement.

**Figure 1:**
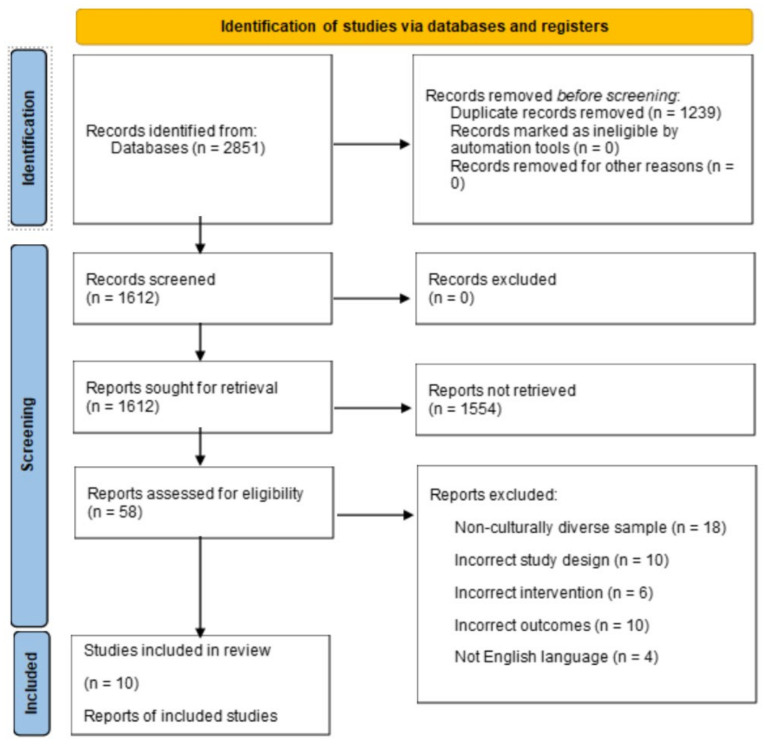
Preferred Reporting Items for Systematic Reviews and Meta-Analyses (PRISMA) flow diagram for study selection. Adapted from Page et al. (2021) [48].

As a result of our initial search, 2851 studies were identified, reduced to 1612 after the removal of duplicates. After reviewing titles and abstracts to determine whether articles were relevant to the scope of the current study, 58 articles remained to be screened in full text. Of the 58 articles screened, an additional 48 were excluded (see Figure 1 for exclusion reasons). The reference lists of the remaining 10 articles were then screened, which revealed no additional article that met criteria for inclusion. A final total of 10 articles were included for review. The studies have been summarized to describe study quality, characteristics and outcomes.

### 2.4. Study Design Characteristics

Among the 10 studies that were included for review, six were conducted in North America, three in Europe and one in Asia. Four of the studies used post-intervention qualitative evaluations. Of the remaining six quantitative studies, three utilized a pre/post-test design without a control group, one utilized a randomized controlled trial design, one a case series design and one utilized a case–control design.

### 2.5. Quality Assessment

Qualitative studies were appraised by two reviewers (L.S. and J.G.) for methodological quality using the Critical Appraisal Skills Programme (CASP) Qualitative Checklist [49]. A value of ‘Yes’,’ No’ or ‘Can’t determine’ was assigned to each requirement. Results of this appraisal are captured in Table 1. Quantitative studies were assessed using the National Heart, Blood and Lung Institute’s (NHLBI) Quality Assessment Tools for Before-After Studies With No Control Group, Controlled Intervention Studies, Case-Control Studies, and Case Series Studies [50]. The findings of this assessment are captured in Table 2.

### 2.6. Sample

Reported sample sizes of included studies ranged from N = 12–792 (M = 253.29), with an approximate total of N = 1950. Two studies [16,51] did not report the sample size. Study samples across the 10 included studies comprised a number of ethnic minority and non-Anglo-Saxon groups including: African American FV (family violence) perpetrators [28], Hispanic/Latina FV perpetrators [16,52,53,54], Canadian Aboriginal FV perpetrators [51,55], Vietnamese FV perpetrators [56], Swedish FV perpetrators [57], and an ‘immigrant’ group which included European, African, American and Asian ethnic groups [58]. Amongst these, three studies [52,56,57] examined non-Anglo-Saxon majority populations, whilst the remaining seven [16,28,51,53,54,55,58], examined sub-ethnic groups within broader populations.

**Table 1:** Quality assessment of included qualitative studies.

Reference (Year)					Criteria						Overall Assessment of Methodological Quality
	1	2	3	4	5	6	7	8	9	10	
Hancock & Sui (2009) [16]	+	+	+	+	+	+	+	+	+	+	No or very minor concerns
Parra-Cardona et al. (2013) [53]	+	+	+	+	+	+	+	+	+	+	No or very minor concerns
Welland & Ribner (2010) [54]	+	+	+	+	+	+	+	+	−	+	No or very minor concerns
Hoang, Quach & Tran (2013) [56]	+	+	+	+	+	+	+	−	+	+	No or very minor concerns

Note. Criteria: 1 = Was there a clear statement of the aims? 2 = Was a qualitative methodology appropriate? 3 = Was the research design appropriate? 4 = Was the recruitment strategy appropriate? 5 = Was the method of data collection appropriate? 6 = Was the relationship between the researcher and participant adequately considered? 7 = Were ethical issues taken into consideration? 8 = Was the data analysis sufficiently rigorous? 9 = Was there a clear statement of the findings? 10 = Was the value of the research discussed? Symbols: + =yes; − = no.

**Table 2:** Quality assessment of included quantitative studies.

Authors (Year)		Criteria														Quality Rating
		1	2	3	4	5	6	7	8	9	10	11	12	13	14	
Gondolf (2004) [14]	Assessed with Quality Assessment Tool for Controlled Intervention Studies (Criteria B)	+	+	−	?	?	+	+	−	+	+	+	+	+	+	Fair
Zellerer (2003) [51]	Assessed with Quality Assessment Tool for Before-After (Pre-Post) Studies with No Control Group (Criteria A)	+	+	+	?	?	+	−	/	?	?	−	−			Fair
Echeburúa (2006) [52]	Assessed with Quality Assessment Tool for Before-After (Pre-Post) Studies with No Control Group (Criteria A)	+	+	+	+	−	+	+	/	+	+	−	/			Good
Puchala et al. (2010) [55]	Assessed with Quality Assessment Tool for Case Series Studies (Criteria D)	+	+	+	+	+	+	+	+	+						Good
Haggård et al. (2015) [57]	Assessed with Quality Assessment Tool of Case Controlled Studies (Criteria C)	+	+	+	+	+	+	/	−	+	+	+	+			Good
Echauri et al. (2013) [58]	Assessed with Quality Assessment Tool for Before-After (Pre-Post) Studies with No Control Group (Criteria A)	+	+	+	+	+	+	+	/	+	+	+	/			Good

Note. Quantitative studies were assessed using the Quality Assessment Tools published by the National Heart, Lung and Blood Institute (NHLBI) [50]. The criteria for each of the utilised tools have been fully explained in Supplementary Materials S3. Symbols: + = yes; − = no; ? = unclear; / = not applicable.

## 3. Results

### 3.1. Culturally Specific Interventions

All studies included for review, with the exception of one [58], evaluated intervention programs delivered in group sessions and not individually. Languages of program delivery included English [28,51,55], Spanish [16,52,53,54,58], Swedish [57], and Vietnamese [56]. In addition to delivery in non-English languages, culturally distinct components of intervention programs also included: the use of culturally knowledgeable facilitators (i.e., those who either spoke the language preferred by the clients, were from the same cultural background, or had training in delivering services to a specific cultural group) [16,28,51,53,55], discussion of culturally diverse expressions of masculinity [16,28,53,55,56], use of cultural healing traditions [52,56], and recognition of cultural issues and challenges faced by the men [16,28,51,53,56].

### 3.2. Measures of Recidivism

Four studies included some measure of violent recidivism or further violence as a metric of program effectiveness in their evaluation of the culturally specific program [28,56,57,58]. Data on reoffending were collected either through self-report [56,58], partner report [28], or government-managed crime registries [57].

### 3.3. Key Findings

#### 3.3.1. Recidivism

The key qualitative and quantitative findings of the studies are presented in Supplementary Materials S2. The table demonstrates that of the 10 included studies, all studies (with the exception of one [57]), showed positive outcomes associated with engagement in culturally specific interventions. Outcome measures varied between the studies, however, six studies [16,28,51,55,56,58] reported either a complete absence or reduction in episodes of abuse. Echauri et al. [57] found that in 85.9% of cases, treatment was effective in reducing physical and psychological perpetration of abuse, with success rates increasing to 87% at the 12-month follow up period. These findings were consistent across immigrant and national populations. Gondolf [28] found that re-assault rates, as reported by the men’s partners, increased from 32% in the first 15 months following treatment, to 42% at 48 months following treatment, which was adjusted to 47% for underreporting using arrest records and men’s self-report. However, when using a retrospective approach from the end of the follow-up period to allow for the intervention to take effect, only 10% of men had re-assaulted their partners in the previous year and over two-thirds of women reported an improvement in their quality of life. Similarly, Hoang et al. [56] reported following engagement with the Responsible Men’s Club, almost 70% of men had not perpetrated any violence, and of the remaining 30% there had been only one episode of violence in the last six months, compared to between two and six episodes in the pre-intervention survey. Of the people that engaged with traditional healing elders (*n* = 76), 49 people reported drastic reductions in domestic violence during their engagement, 9 reported no change in the violence, and 11 reported a continued escalation in violence or they were lost to follow up. 29 of the 49 individuals reported that the violence had completely stopped. Qualitative findings from both Hancock and Siu [16] and Zellerer [51] indicate that men who engaged in the intervention programs believed the programs had helped them to understand and control their violence [51] and they were able to provide suggestions to other participants on how to avoid abusive strategies [16]. However, these results do not in themselves indicate a reduction in violence.

#### 3.3.2. Psychopathology

An improvement in psychopathological symptoms as a result of engaging in culturally specific intervention programs were reported by six studies [16,51,52,54,55,58]. Echauri et al. [58] defined treatment success as a complete disappearance of abuse and a reduction in psychopathological symptoms. Pathological symptoms were measured using the Symptom Checklist 90 Revised (SCL-90-R) and State-Trait Anger Expression Inventory-2 (STAXI-2) at pre and post and at a 12-month follow-up. The SCL-90-R is a self-report measure of psychopathology that includes measures of depression, anxiety, hostility, psychosis, somatization, obsessive compulsive behavior, interpersonal sensitivity, phobic anxiety, and paranoid ideation [59]. The STAXI-2 measures the intensity of state and trait anger expression. Echauri et al. [58] reported significant improvement in all of the variables for both immigrant and national samples, with most participants further improving between posttreatment and the 12-month follow-up. Similarly, Echeburua et al. [52] reported an overall reduction in psychopathological symptomatology in the posttreatment assessment, as measured in the SCL-90-R and a reported increase in overall emotional stability. In the Puchala et al. study [55], pre and post assessment of psychopathological complaints including anxiety, fear, sleep problems and sadness, indicated a reduction in overall distress as assessed by the MYMOP2 (Measure Your Medical Outcome Profile 2) measure. Self-reported reductions in anxiety were also identified in Welland and Ribner [54], and a reduction in aggressiveness was observed by staff among participants in the Zellerer study [51].

#### 3.3.3. Gender-Related Attitudes

A change in cognitive biases and beliefs about women and gender roles, were reported in four studies [16,52,54,56]. Echeberua et al. [52] reported a lower score on The Inventory of Distorted Thoughts About Women at the posttreatment assessment, indicating fewer cognitive distortions in relation to men’s attitudes toward women. Similarly, a pre and posttreatment measure in the Hoang et al. study [56] indicated that men’s attitudes towards gender roles and masculine identities had improved significantly. In addition, men reported learning from thinking about situations from their partners points of view and their marital relationships. Similarly, participants in the Welland and Ribner study [54], discussed a shift in their attitudes towards gender roles, including a recognition of their partners rights and agency, which they reported as positive for both them and their partners. Most participants reported the concept of gender equality as new to them and all participants appeared to accept new ideas about gender roles. A participant in the Hancock and Siu study [16] reported that engagement in the program had helped to change his view of women from objects to persons.

#### 3.3.4. Family Communication

Improvements in communication and alternative coping strategies to the use of violence were discussed in four of the studies [16,51,54,56]. In two of the studies [54,56], improved communication and strategies also supported improved parenting styles, and in one study [51], communication among peers was observed to improve during the treatment. Improved communication styles included ‘time-out’ strategies to help men recognise the changes in their emotions and physical state when entering conflict [56]; making ‘I rather than you’ statements to express their feelings in a non-violent manner [56]; using role plays to practice conflict resolutions [16]; and learning to be honest, developing respectful communication skills and to communicate with other men [54].

#### 3.3.5. Program Satisfaction

Participant satisfaction with engaging in interventions was reported as an outcome in four of the studies [51,52,53,54]. Reports of satisfaction varied, with qualitative studies indicating that satisfaction was derived from an ability to explore their cultural and spiritual heritage [51], learning how to be nurturing fathers [54], and establishing close interpersonal relationships between participants and group facilitators [53]. Echeburua et al. [52] incorporated The Questionnaire of Satisfaction with Treatment measure. However, no pre or post measures were provided. Participant satisfaction was instead assumed through the high levels of engagement, with 92% completing the program. Three other studies reported completion rates as an outcome of the intervention program [16,28,57].

One study found no positive outcomes associated with the interventions [57]. In this study, the rates of violent recidivism were higher among the treatment group in comparison to the control group, and rates of intimate partner violence recidivism were consistent across the two groups.

## 4. Discussion

This systematic review shows that there is hope for behavior change to occur through culturally relevant male perpetrator intervention programs for intimate partner violence. This is the first systematic review to methodically examine the current evidence in relation to the effectiveness of male behavior modification programs for domestic violence offenders that have incorporated a ‘cultural’ aspect or have included clients from ethnic minority and/or non-Anglo-Saxon backgrounds. Our review examined features of intervention programs that determine their unique applicability to ethnic minority populations and outcomes relevant to behavior change, a reduction in recidivism, improved mental health, and client introspection of transformations of gender related attitudes.

While the outcome measures varied, most studies in this review showed positive outcomes as a result of perpetrators’ engagement with the culturally specific intervention. The men in 6 [16,28,51,55,56,58] of the 10 studies did not repeat the overall abuse or reduced their incidences of abuse; these findings are in contrast to previous studies [11,24,25] that found treatment programs had almost a nil effect on recidivism. It is to be noted that in these past studies, participant attrition from the from was high that prevented any distinct conclusions being drawn about program effectiveness. Furthermore, one study [58] in the present review demonstrated effectiveness in reducing physical and psychological abuse even at the 12-month follow up period. However, not all studies though demonstrated long-term improvements. Previous studies that offered group treatments to IPV perpetrators had limited success in reducing the cycle of violence, thus demonstrating the importance of individualized, tailored intervention approaches.

Upon close examination of the specific aspects of the reviewed programs that may have led to successful outcomes, we found that programs implemented in languages relevant to the client group were fundamental to their success; these programs also benefited from bi-cultural facilitators. They also specifically measured recidivism to determine the role of their program in addressing re-offending behavior. It is recommended that other programs also incorporate such aspects and monitor clients’ behavior over the long term to succeed. These findings align with intervention program objectives to address recidivism.

The review shows that cultural engagement could enhance client participation and reduce their attrition, a problem highlighted by Chang and Saunders [26] among minority groups. For example, Hoang et al. [56] enabled clients to interact with traditional healing elders; they believed that increased engagement with community leaders from the community could be one reason for a reduction in domestic violence in over half the participants. While previous studies have not specifically examined the influence of traditional healing elders, the importance of practitioners applying techniques addressing the social and cultural contexts have been explored [10]. We, however, need to further understand the training required for traditional healers to be part of behavior modification programs and appraise their pre-existing notions of gender equality and issues relevant to reducing violence against women prior to involving them in intervention programs.

Studies [16,51,52,54,55,58] that showed successful treatment of clinical symptoms, including improvements in psychopathology, anger, emotional stability, distress, and anxiety, had evaluated programs that had the following defining cultural features: diversity in language/s used, facilitators training cultural nuances or facilitators from the same background or a combination of these with cultural healing traditions [55]. These findings reiterate the guidance provided by Almeida and Dolan-Delvecchio [32] that program practitioners should be culturally sensitive and that treatment systems are culturally comprehensive and not only developed and implemented with dominant, White-centric theories.

Fewer than half the studies reviewed found alterations in thinking patterns and beliefs about women and gender norms. However, those [16,52,54,56] that were successful focused on adopting culturally relevant strategies to address masculinity and promote gender equal attitudes [56]. Empowerment, usually used to facilitate women’s growth after a negative event, was recognized as an essential component of behavior modification by Hoang et al. [56] who indicated that men could use their agency to challenge traditional notions of unequal power relations and deconstruct and reconstruct their notions of masculinity. They applied this framework to their participants in Vietnam and asked men to re-think their traditional social roles and what it meant to be a man in the Vietnamese society. This study showed success in creating alternative narratives for men by using existing narratives and using culturally appropriate knowledge to deconstruct them. Similarly, Welland and Ribner [54] demonstrated that when Latino participants in their study realized that being *machista*, a term commonly used in Latin American cultures to describe male chauvinist behavior, was not useful in the context of their personal relationships, they changed their attitudes towards gender roles. These align with some aspects of the Duluth model that uses a feminist approach to challenge men’s sense of power and control and educates them with alternative skills to reduce conflict in their relationships and their violence towards their partners [14]. Thus, the model adopted by Hoang et al. [56] and Welland and Ribner [54], when well-resourced and applied in a culturally relevant way in each cultural context, could lend itself to a program to advance practitioners’ understanding of cultural norms and address perpetrators’ behaviors successfully [32].

This review also shows that perpetrator behavior modification programs could assist with improved communication skills. Four studies [16,51,54,56] demonstrated that their clients were able to communicate better to their partners/ex-partners and peers after intervention. They conducted their program in a language suitable to their client group and addressed challenges faced by the men that were relevant to their cultural background and demographic characteristics. For example, Welland and Ribner [54] addressed the importance of *respeto*, which is traditionally regarded as respectful behavior to high status individuals; participants in their program identified the importance of this mannerism of communication in an intimate relationship. Participants recognized that they had previously lacked empathetic communication in their personal relationships due to the male socialization in their society, and therefore had not been exposed to childhood experiences of positive role models [54]. Removing language barriers therefore could lead to an improvement in relationship transactional skills. This could also pave the way for better parenting skills [54].

Another outcome measured in four studies [51,52,53,54] was client satisfaction with program engagement. These were mainly introspective measures. Those programs that had high levels of engagement such as discussing participants’ cultural and spiritual heritage, learning to be develop their parenting skills, or where a close interpersonal relationship was fostered between participants and facilitators led to lower client attrition; this outcome is essential as previous studies [10,26] have shown a high participant attrition, especially those from minority communities, from perpetrator intervention programs. Thus, when perpetrators of IPV are engaged and continue to participate, they are more likely to benefit from the program and can modify their behavior.

### Limitations

There are however caveats to the positive findings illustrated in this review. First, this review could only find 10 studies that met the inclusion criteria; most of these were conducted in North America and they examined various minority ethnic and non-Anglo-Saxon groups. Therefore, the applicability of the present findings to the multicultural diversity in this world, is limited. There was also only one study that examined an Indigenous group; this underscores the necessity of examining the effectiveness of domestic violence perpetrator intervention programs for other Indigenous groups. Nonetheless, there are some cultural norms that are similar across several minority groups, therefore, the findings would still have applicability. Another limitation is that success of the perpetrator intervention program was measured in a variety of ways and not consistently across the studies. This allows for minimal integration of the factors that could lead to the determination of an efficacious program. Future studies should incorporate some common measures of behavioral outcomes and evaluate them in the short and long-term. A main limiting factor that reduces the deduction of the findings is that most studies examined perpetrators’ reports of successful outcomes and not the men’s partners’ perspectives of behavior change, reduced recidivism, or demonstration of gender-related attitudes within the family. Without such an examination, it is difficult to ascertain whether participants and program facilitators have the same introspection and observation of behavioral changes that those who were abused consider. Future studies must make an effort to assess changes through other external assessments.

## 5. Conclusions

Despite the limitations, this review is valuable in providing a systematic overview of what works for perpetrators from minority ethnic groups to alter their behavior for the better through behavior modification intervention programs. We have demonstrated the factors that lead to successful outcomes in male domestic violence perpetrator intervention programs including: client-associated language programs, facilitators who are culturally trained and/or bicultural facilitators who have an advanced knowledge of the cultural norms of the client group, developing and implementing programs that suit the cultural background of the clients (with an in-depth understanding of cultural norms and nuances relevant to interpersonal communication), discussion of patriarchal norms such as *machismo* that underpin and reinforce male domination and female subordination, discussions of gender roles and attitudes to gender equality specific to each cultural context; and greater culturally relevant client engagement. With the incorporation of such culturally relevant strategies, the studies included in this review have demonstrated that positive outcomes including improved mental health, reduced recidivism, behavior change, and better attitudes to gender equality could be achieved for perpetrators and their families.

This review thus contributes to the body of literature that examines the effectiveness of male domestic violence perpetrator intervention programs. It has examined the unique and universal factors relevant to diverse cultural groups that could assist with improved outcomes to reduce violence against women. Further research is required to examine what works for different cultural groups. Dominant White-centric models should not be applied to perpetrator clients who are from minority ethnic backgrounds or from non-Anglo-Saxon groups. Rather, program developers and facilitators could recognize the cultural nuances of their client group and then incorporate the successful approaches utilized by the studies examined in this review. This will lead to greater client engagement and a possibility of success. This will ultimately lead to men altering their maladaptive behaviors and making relationships safer for women and their families.

## Data Availability

Not applicable.

## Supplementary Materials

The following supporting information can be downloaded at: https://www.mdpi.com/article/10.3390/ijerph192215180/s1, Supplementary Materials S1, Systematic Review Search Strategy; Supplementary Materials S2, Table S1: Methodological characteristics and key findings of the included studies; Supplementary Materials S3, Criteria for the Quality Assessment Tools (NHLBI).
