# Supplementary material for: The Effectiveness of Culturally Specific Male Domestic Violence Offender Intervention Programs on Behavior Changes and Mental Health: A Systematic Review
Source: Int J Environ Res Public Health. 2022 Nov 17;19(22):15180. doi: 10.3390/ijerph192215180 (PMC9690755; doi:10.3390/ijerph192215180)
Supplement: Supplementary file 1 [file ijerph-19-15180-s001.zip › ijerph-1949629-supplementary.pdf]

International Journal of
*Environmental Research and Public Health*

MDPI

**Supplementary Materials S1**

*Systematic Review Search Strategy.*

The following databases were searched on five occasions between June 2017 and August 2022.

| Platform | Database |
|---|---|
| EbscoHost | Academic Search Complete |
| EbscoHost | AMED – The Allied and Complementary Medicine Database |
| EbscoHost | CINAHL Complete |
| EbscoHost | Criminal Justice Abstracts with Full Text |
| EbscoHost | E-Journals |
| EbscoHost | Health Source: Nursing/Academic Edition |
| EbscoHost | PubMed |
| EbscoHost | APA PsycARTICLES |
| EbscoHost | APA PsycEXTRA |
| EbscoHost | Psychology and Behavioural Sciences Collection |
| EbscoHost | APA PsycINFO |
| EbscoHost | Social Work Abstracts |

The following subject headings and keywords were used to conduct the searches:

PSYCINFO

| Concept | Subject headings | Additional keywords |
|---|---|---|
| 1. Culturally diverse | Cross Cultural Psychology<br>Cultural Sensitivity<br>Racial and Ethnic Groups<br>Sociocultural factors<br>Cross Cultural Communication | Cultur* OR rac* OR ethnic* OR migra* OR immigra* OR CALD OR cultur* linguistic* divers* OR refugee* OR asylum seek* |
| 2. Offenders | Antisocial behaviour<br>Crime<br>Perpetrators | Perpetrat* OR offend* OR batterer OR aggress* |
| 3. Family violence | Domestic violence | Family violen* OR domestic violen* OR intimate partner violen* OR IPV OR spous* abuse OR partner abuse OR domestiv abuse OR battered wom?n OR marital violen* inter#partner violen* |
| 4. Intervention | Intervention<br>Rehabilitation<br>Treatment<br>Program evaluation | Interven* OR Program* OR therap* OR trial |

PSYCINFO

exp "Cross Cultural Psychology"/ OR exp "Cultural Sensitivity"/ OR exp "Racial and Ethnic Groups"/ OR exp "Sociocultural factors"/ OR exp "Cross Cultural Communication"/ OR Cultur* OR race OR racial OR ethnic* OR migra* OR immigra* OR CALD OR "culturally and linguistically diverse" OR refugee* OR asylum seek*
exp "Antisocial behaviour" OR exp "Crime"/ OR exp "Perpetrators"/ OR Perpetrat* OR offend* OR batterer OR aggress*
exp "Domestic violence"/ OR "Family violence" OR "domestic violence" OR "intimate partner violence" OR IPV OR "spousal abuse" OR "spouse abuse" OR "partner abuse"

OR “domestic abuse” OR “battered woman” OR “battered women” OR “marital violence” OR “inter-partner violence” OR “inter partner violence”
exp “Program evaluation”/ OR exp “Therapeutics”/ OR Interven* OR Program* OR therap* OR trial

PUBMED

| Concept | MESH terms | Additional keywords |
| --- | --- | --- |
| 1. Culturally diverse | Culture<br>Ethnic Groups | Cultur* OR rac* OR ethnic* OR migra* OR immigra* OR CALD OR cultur* linguistic* divers* OR refugee* OR asylum seek* |
| 2. Offenders | Criminals | Perpetrat* OR offend* OR batterer OR aggress* |
| 3. Family violence | Domestic Violence<br>Intimate Partner Violence | Family violen* OR domestic violen* OR intimate partner violen* OR IPV OR spous* abuse OR partner abuse OR domestiv abuse OR battered wom?n OR marital violen* inter#partner violen* |
| 4. Intervention | Program Evaluation<br>Therapeutics | Interven* OR Program* OR therap* OR trial |

PUBMED

“Culture”[Mesh] OR “Ethnic Groups” [Mesh] OR Cultur* OR race OR racial OR ethnic* OR migra* OR immigra* OR “CALD” OR “culturally and linguistically diverse” OR refugee* OR “asylum seek*”
“Criminals”[Mesh] OR Perpetrat* OR offend* OR batterer OR aggress*
“Domestic Violence”[Mesh] OR “Intimate Partner Violence”[Mesh] OR “Family violen*” OR “domestic violen*” OR “intimate partner violen*” OR “I?P?V” OR “spousal abuse” OR “spouse abuse “ OR “partner abuse” OR “domestic abuse” OR “battered woman” OR “battered women” OR “marital violen*” OR “inter#partner violen*”
“Program Evaluation”[Mesh] OR “Therapeutics”[Mesh] OR program OR programs OR Interven* OR therap* OR trial

**Supplementary Materials S2**

**Table S1.** Methodological characteristics and key findings of the included studies.

| Author | Country | Sample size | Sample characteristics | Study Design | Measures/Assessments | Intervention | Key Findings |
|---|---|---|---|---|---|---|---|
| Echauri, et al. (2013) | Spain | *N* = 300 Spanish citizen (*n* = 150) Immigrant (*n* = 150) | Age: *M* = 37.50, *SD* = 9.33.<br><br>Education: primary (*n* = 177, 59%), secondary (*n* = 110, 36.6%), university (*n* = 13, 4.3%)<br><br>Employment: employed (*n* = 180, 60%), unemployed (*n* = 109, 36.3%), retired (*n* = 11, 3.7%)<br><br>Children: yes (*n* = 195, 65%), no (*n* = 105, 35%)<br><br>Nationality: Spanish (*n* = 150, 50%), South America (*n* = 230, 76.6%), Africa (*n* = 34, 11.3%), Europe (*n* = 32, 16.6%), Asia (*n* = 4.3%). | Prospective ex post facto design, two groups. | General Structured Interview of Batterer Men.<br><br>Symptom Checklist-90-Revised (SCL-R-90).<br><br>State-Trait Anger Expression Inventory (STAXI-2).<br><br>Measures were obtained pre- and post-treatment and at a 12 month-follow up. | 20 one-hour individual program sessions, based on cognitive behavioural principles from the Echeburua (2006) pilot programme.<br><br>Sessions 1–3: Motivational aspects<br><br>Sessions 4–15: Treatment of the psychopathological symptoms<br><br>Sessions 16–20: Relapse prevention | Success rate post-treatment: Immigrant group 34.6%. Spanish citizen group 34.6%. Improvement rate post-treatment: Immigrant group 51.3%. Spanish citizen group 51.3%. Failure rate post-treatment: Immigrant group 14%. Spanish citizen group 14%.<br><br>Success rate at follow-up: Immigrant group 35.3%. Spanish citizen group 38.6%. Improvement rate at follow-up: Immigrant group 52%. Spanish citizen group 48%. Failure rate at follow-up: Immigrant group 12.6%. Spanish citizen group 13.3%. Group comparison: $\chi^2 = 0.49$ at follow-up.<br><br>SCL-R-90-GSI: $F = 19.2$, $p < .001$<br><br>STAXI-2: $F = 11.4$, $p < .001$ |
| Echeburúa (2006) | Spain | *N* = 52 | Adult males<br>Age: 18–65 years, *M* = 40 | Quantitative pre/post-test design. | Inventory of Distorted Thoughts About Women.<br><br>Inventory of Distorted Thoughts on the Use of Violence. | 20 one-hour individual program sessions. Based on cognitive behavioural principles. | Cognitive bias about women: $t = 2.82$, $p < .01$.<br><br>Cognitive bias about the use of violence: $t = 2.67$, $p < .01$. |

| | | | | | | | |
|---|---|---|---|---|---|---|---|
| | | | Serving a sentence for a serious offence related to gender violence. Education: 81% left school at minimum age. 4% university educated. | | Interpersonal Reactivity Index.<br>SCL-90-R.<br>STAXI.<br>Barratt Impulsiveness Scale.<br>Self-Esteem Scale.<br>Inadaptation Scale.<br>Scale of Expectation of Change.<br>Questionnaire of Satisfaction with Treatment. | Sessions 1–3: Motivational aspects<br>Sessions 4–15: Treatment of the psychopathological symptoms<br>Sessions 16–20: Relapse prevention | SCL-90-R-GSI: $t = 2.01$, $p < .05$.<br>Empathy: $t = 1.01$, $p > .05$.<br>State-anger: $t = 2.26$, $p < .05$.<br>Impulsivity: $t = 0.53$, $p > .05$.<br>Self-esteem: $t = 0.52$, $p > .05$.<br>Inadaptation: $t = 1.55$, $p > .05$. |
| Gondolf (2004) | USA | $N = 503$ | African-American males arrested for domestic violence in Pittsburgh.<br>Unemployment (40%)<br>Education (27% with some college)<br>Unmarried (70%) | Randomised controlled trial. | Short Michigan Alcoholism Screening Test.<br>Racial Identity Attitude Scale. | Minimum 16 weekly group sessions. Included basic skills and reasons for stopping violence, cultural issues relevant to African American men and masculinity, increased use of concrete examples, vignettes, and directive questions. | Completion: 52–57%. Men with high cultural identification - culturally focussed (CF): 63%, conventional with African American group (AA): 65%, conventional with mixed-ethnicity group (MX): 40%, $p < .05$.<br>Reoffending: CF: 24%, AA: 19%, MX: 25%. Men with high cultural identification: CF: 15%, AA: 19%, MX: 31%, *n.s.* |
| Haggard et al. (2015) | Sweden | $N = 792$ | Intervention group<br>Age: $M = 38.9$, $SD = 10.3$, range = 19–66.<br>Prison sentence: $M = 255$ days, $SD = 157$<br>Control group | Quantitative case-controlled group | Spousal Assault Risk Assessment guide (SARA).<br>Alcohol Use Disorder Identification Test (AUDIT). | Manual-based treatment program originating from the Duluth Domestic Abuse Intervention Project. Changed language from English to Swedish, modernized remakes of | 73% ($n = 249$) of participants completed the Integrated Domestic Abuse Program (IDAP) treatment program. Attendance rate was ($M = 27$, $SD = 4.99$) for group sessions and |

| | | | | | | | |
|---|---|---|---|---|---|---|---|
| | | | Age: *M* = 40.2, *SD* = 11.1, range = 19–75. Prison sentence: *M*: 130, *SD* = 84 | | Drug Use Disorder Identification Test (DUDIT). Reconviction data obtained from the National Crime Register. | original video clips in Swedish which were used to illustrate problem behaviour and stimulate discussion. 27 offender group sessions at 2 hours and at least 8 individual sessions. | (*M* = 13, *SD* = 14.39) for individual sessions. Violent recidivism for treatment group was 25% (*n* = 84) while for the control group, 23% (*n*= 104). Rate of IPV recidivism for the IDAP treatment group and control group was 19% (*n* = 65, *n* = 84 respectively) in total. |
| Hoang, Quach & Tran (2013) | Vietnam | *N* = 36 | Male perpetrators aged in their 30s–50s. | Qualitative design with pre-post evaluation of program. | In-depth interviews conducted with perpetrators pre and post involvement with the programme. Included open and closed questions about couple relationships; children; form, frequency, and context of violence. Mid-term in-depth interviews were conducted with four Club members to get insight into men's interaction in the Club sessions and activities. Pre- and post-Club surveys were completed by all participants and examines; history of perpetrators; knowledge, attitude, and practices regarding gender equality, sexuality and violence. Developed specifically to examine themes explored in the club. | 14 workshop sessions that discussed gender norms and values, family conflicts, violence, anger management, sexual relationship, and fatherhood. Materials adapted from RESPECT. | Gender attitudes: Average score at pre-test = 9.1 and post-test = 6.16 (maximum score = 10). Sexuality perception: Average score at pre-test = 9 and post-test = 14.2 (maximum score = 20). Peaceful and comfortable discussions with wives during tension and conflict increased. Pre-test: 31.3%, Post-test 66.7%. 70% of perpetrators stated no further occurrence of violence. 30% stated one episode of violence. |

| | | | | | | | |
|---|---|---|---|---|---|---|---|
| Parra-Cardona et al. (2013) | USA | *N* = 21 | Latino men. Origin: Mexican (*n* = 20), Puerto Rican (*n* = 1). Age: *M* = 36.43 Years in America: *M* = 17.36, range = 5 to 38. Relationship status: relationship (*n* = 16). Education: Elementary school (*n* = 5), high school (n = *10*), college (*n* = *6*). Income: lower than $10,000 (*n* = 2), $10,000 – $20,000 (*n* = 8), $31,000 - $40,000 (*n* = 9), $41,000 - $50,000 (*n* = 1), higher than $60,000 (*n* = 1). | Qualitative design. | Qualitative interviews on reason for program referral and experience of the program. | The Raíces Nuevas (New Roots) intervention. A culturally informed version of the Spanish version of the Duluth curriculum 'Creating a Process of Change for Men Who Batter'. Curriculum was revised to include idiomatic expressions and adapted to address common challenges experienced by Latino men in the Midwest. | Results suggest that participation in the Raíces Nuevas batterer intervention program may be beneficial for Latino immigrant men.<br><br>For the core concept and each category men perceived the intervention as successful. |
| Hancock & Siu (2009) | USA | *N* = 12–18 | Mexican men from rural settings. Years in America: Majority for less than 5 years. Some less than 1 year. Majority undocumented. Socioeconomic status: Majority working class. Education: Majority low. English skills: Majority low. Abuse type: mild, moderate, severe. 'situational and intergenerational' abusers | Qualitative observation study. | Observations and feedback. | 26-week program of 90-minute sessions weekly with 12 to 18 members. Sessions conducted in Spanish. | Exploration allowed men to alleviate feelings of oppression, anger, anxiety, fear, helplessness, frustration, loss and grief, and other negative responses to the immigration experience.<br><br>Men engaged more actively and positively than in the Duluth intervention.<br><br>Almost 90% completion rate with a less than 25% re-enrolment rate. |

| | | | | | | |
|---|---|---|---|---|---|---|
| Puchala et al. (2010) | Canada | $N = 69$ | Multicultural group: Canadian Aboriginal. Community: Salteaux ($n = 8$), Dakota ($n = 7$), Dene ($n = 18$), Aboriginals from out of province ($n = 10$), Cree ($n = 26$). | Clinical case series. | My Medical Outcomes Profile 2 (MYMOP-2). Participants identify their two most problematic symptoms and rate the severity on a scale of 0–6. | Elders invited to participate in clinical contacts. Elder contact involved prayer, storytelling, talking circle discussion, sweat lodge ceremonies, pipe ceremonies, shaking tent ceremonies, involvement of family, discussion of aboriginal masculinities, reconstruction of self-narratives. | MYMOP-2 symptom severity: $p < 0.0001$ |
| Welland and Ribner (2010) | USA | $N = 159$ (Interviews with $n = 12$) | Latino men living in South California. Mexican origin: 89% Aged 35 years or younger: 35% Had a 6th grade education or less: 37% Had incomes below the federal poverty line: 70% | Qualitative program evaluation. | Interview focusing on protective and risk factors, strengths of Latino culture, treatment implications and techniques for Latinos. | 52-week treatment program.<br><br>Focusing on effective parenting skills for men; gender roles; discussion of discrimination towards immigrants and women; immigration and changing gender roles; marital sexual abuse; and spirituality as related to violence prevention. | Participants report new attitudes towards gender roles, improved communication and respect, increased responsibility for their behaviour, reduced anxiety, and improved approaches to parenting. |
| Zellerer (2003) | Canada | Unspecified | Multicultural group: Canadian Aboriginal. Majority < 30 years old (20–41 years) Sentence length: $M = 5$ (2–25 years). | Qualitative program evaluation. | Interview about participant satisfaction. | 29 sessions, 2.5 hours long over 4 months.<br><br>Contemporary therapy methods combined with traditional Aboriginal teachings. Spiritual and traditional teachings, using the medicine wheel, were incorporated into the program. | High expectations of the program reducing violence were met according to participant report. Participants reported high satisfaction with the program. Preferred components were talking and sharing in group discussions as well as cultural ceremonies. Participant reports corroborated by facility staff. |

**Supplementary Materials S3**

*Criteria for the National Heart, Lung and Blood Institute Quality Assessment Tools.*

**Criteria A**: 1 = Was the study question or objective clearly stated? 2 = Were eligibility/selection criteria for the study population prespecified and clearly described? 3 = Were the participants in the study representative of those who would be eligible for the test/service/intervention in the general or clinical population of interest? 4 = Were all eligible participants that met the prespecified entry criteria enrolled? 5 = Was the sample size sufficiently large to provide confidence in the findings? 6 = Was the test/service/intervention clearly described and delivered consistently across the study population? 7 = Were the outcome measures prespecified, clearly defined, valid, reliable, and assessed consistently across all study participants? 8 = Were the people assessing the outcomes blinded to the participants' exposures/interventions? 9 = Was the loss to follow-up after baseline 20% or less? Were those lost to follow-up accounted for in the analysis? 10 = Did the statistical methods examine changes in outcome measures from before to after the intervention? Were statistical tests done that provided p values for the pre-to-post changes? 11 = Were outcome measures of interest taken multiple times before the intervention and multiple times after the intervention (i.e., did they use an interrupted time-series design)? 12 = If the intervention was conducted at a group level (e.g., a whole hospital, a community, etc.) did the statistical analysis take into account the use of individual-level data to determine effects at the group level?

**Criteria B**: 1 = Was the study described as randomized, a randomized trial, a randomized clinical trial, or a randomized controlled trial (RCT)? 2 = Was the method of randomization adequate (i.e., use of randomly generated assignment)? 3 = Was the treatment allocation concealed (so that assignments could not be predicted)? 4 = Were study participants and providers blinded to treatment group assignment? 5 = Were the people assessing the outcomes blinded to the participants' group assignments? 6 = Were the groups similar at baseline on important characteristics that could affect outcomes (e.g., demographics, risk factors, co-morbid conditions)? 7 = Was the overall drop-out rate from the study at endpoint 20% or lower of the number allocated to treatment? 8 = Was the differential drop-out rate (between treatment groups) at endpoint 15 percentage points or lower? 9 = Was there high adherence to the intervention protocols for each treatment group? 10 = Were other interventions avoided or similar in the groups (e.g., similar background treatments)? 11 = Were outcomes assessed using valid and reliable measures, implemented consistently across all study participants? 12 = Did the authors report that the sample size was sufficiently large to be able to detect a difference in the main outcome between groups with at least 80% power? 13 = Were outcomes reported or subgroups analyzed prespecified (i.e., identified before analyses were conducted)? 14 = Were all randomized participants analyzed in the group to which they were originally assigned, i.e =, did they use an intention-to-treat analysis?

**Criteria C**: 1 = Was the research question or objective in this paper clearly stated and appropriate? 2 = Was the study population clearly specified and defined? 3 = Did the authors include a sample size justification? 4 = Were controls selected or recruited from the same or similar population that gave rise to the cases (including the same timeframe)? 5 = Were the definitions, inclusion and exclusion criteria, algorithms or processes used to identify or select cases and controls valid, reliable, and implemented consistently across all study participants? 6 = Were the cases clearly defined and differentiated from controls? 7 = If less than 100 percent of eligible cases and/or controls were selected for the study, were the cases and/or controls randomly selected from those eligible? 8 = Was there use of concurrent controls? 9 = Were the investigators able to confirm that the exposure/risk occurred prior to the development of the condition or event that defined a participant as a case? 10 = Were the measures of exposure/risk clearly defined, valid, reliable, and implemented consistently (including the same time period) across all study participants? 11 = Were the assessors of exposure/risk blinded to the case or control status of participants?

12 = Were key potential confounding variables measured and adjusted statistically in the analyses? If matching was used, did the investigators account for matching during study analysis?

**Criteria D**: 1 = Was the study question or objective clearly stated? 2 = Was the study population clearly and fully described, including a case definition? 3 = Were the cases consecutive? 4 = Were the subjects comparable? 5 = Was the intervention clearly described? 6 = Were the outcome measures clearly defined, valid, reliable, and implemented consistently across all study participants? 7 = Was the length of follow-up adequate? 8 = Were the statistical methods well-described? 9 = Were the results well-described?
